# Editorial Chit-Chat

**Published:** 1872-07

**Authors:** 


### EDITORIAL CHIT-CHAT.

PERSONAL.—During our recent visit to Indianapolis, Ind., we had the pleasure of meeting Dr. Parvin and Dr. Bigelow, two highly educated and cultured physicians, whom it is an honor to know.

BABIES.—They grow plenty, numerous in fact, upon the sidewalks, and on the front porches. Our cold weather has kept the little darlings within doors so long, that now, when warm weather has really come, they seem to be swarming. Admiring mamas and fond papas may be seen congregated in little squads upon the walks these pleasant evenings, counting teeth and relating the wonderful exploits of their respective little ones. Listening to their conversation one would suppose that baby is the only one who does things "on the square."

CEREBRO-SPINAL MENINGITIS.—This disease has been very prevalent all over the United States. It has been very fatal in the Western country, so much so, that the inhabitants of Indiana have named it, "cerebro-spinal-cum-and-get-us."

ROB'T F. STOBO, formerly of this city, has gone into business for himself at 52 Maiden Lane, New York. He deals in and imports dye-stuffs, pure chemicals, etc. He has had large experience in the business, is an accomplished gentleman, and deserves success.

THE BINGHAMTON INEBRIATE ASYLUM.—Dr. Turner, the founder of this institution, has written Gov. Hoffman, saying that he will raise by subscription, $66,000 to pay the bonds now due, and will give $5,000 out of his own pocket, provided Drs. Butler, Ray and Nichols be appointed Commissioners to organize the concern.

IMITATION OF HUMAN HAIR.—A patent has been taken out for converting goat's hair into hair for ladies' use. It is said that the hair manufactured from this material cannot be distinguished from human hair. If women expect to continue the wearing of a bushel or so of stuff upon their heads, we are glad they have found a substitute for that with which they now adorn their silly pates.

GUM CHEWING.—Did you ever see a fashionably dressed young damsel upon the street, with a cud of gum in her mouth, and doesn't she appear stupid—not to say idiotic? We see it stated in an exchange that there exists a young woman somewhere in Vermont, who can chew gum in seven different languages, with both eyes shut, and one hand tied behind her back. Our girls have not quite reached that perfection of the art.

A MONSTER BALLOON.—Mr. Sam'l A. King, the celebrated Boston aeronaut, who made an ascension from this city last summer, and with whom we made an ærial voyage, has recently constructed a balloon of mammoth proportions, which is to be used this summer in a series of scientific investigations in the ærial regions. The body of the balloon is a perfect sphere, its circumference one hundred and ninety-one feet, and its capacity nearly one hundred thousand cubic feet. Twelve hundred yards of forty-inch best Lyman mills cloth, furnished expressly for the purpose, were required in its construction. A dozen sewing machines were constantly employed for five months, doing the sewing, and four barrels of oil-varnish were required to cover the monster when completed. This monster balloon, the largest in the world, is named the "Colossus," and expects to make its trial trip, from Boston Common, on the 4th of July.

RASCALLY.—There is a medicine prepared by Dr. Samuel B. Collins, of Laporte, Ind., and extensively advertised throughout the country, as an "Unfailing and Painless Remedy against the Opium Habit," the price being $16 per bottle. A recent analysis of the liquid revealed a syrup, colored with fuchsine, and containing a large proportion of *morphine*. That's a pretty preparation to offer a victim of opium eating, as a cure for the habit.

STARTLING ANNOUNCEMENT.—We have the positive assurance of our friend, S. C. Taber, Esq., who is everywhere known as the former Agricultural Editor of the *Advertiser*, that should he not accept a piscatorial engagement at Fire Island this summer, he will open an agricultural department in the BISTOURY. Those who contend that Sam "don't know beans" about agriculture, will then be convinced to the contrary. At present Mr. Taber is earnestly engaged in erecting a stately mansion near Lake Eldridge, at a cost of several millions of dollars. We have not learned whether he expects to occupy the building himself or not.

PLEASANT VALLEY WINE CO.—In company with Mr. E. F. DIXEY and Mr. GEO. COHEN, of Philadelphia, we recently made an excursion to the wine cellars of the above mentioned company, located near Hammondsport, N. Y. We are indebted to the Superintendent, Mr. C. D. CHAMPLIN, for a very pleasant visit, in conducting us through this extensive establishment.—Also, to Mr. JULES MASSON, who superintends the manufacture of the wine, for explanations and illustrations of the minutia of the manufacture of sparkling wine. The "Carte Blanche," and "Great Western" brands of champagne, manufactured by this company, are becoming famous throughout the country, and are justly regarded as equal, and in many instances superior to many brands imported from abroad.—Two millions pounds of grapes were employed in the manufacture of wines at this cellar alone, last year, and yet Mr. Champlin is unable to supply the demand for his champagne. We are glad to chronicle this evidence of the discrimination of the public between wholesome, pure wines, and the adulterated, inferior stuff, shipped us from abroad.

PRODUCING SLEEP IN INFANTS.—A gentleman writing to the Dublin Quarterly Journal of Medicine, tells how the women of the Himalayas put their babies to sleep. When the mother goes into the field to work, she selects a sheltered spot near a stream, in which she places some straw for the infant's bed. She then directs, by means of a piece of split bamboo, a current of water, of from one to two inches in diameter, on its uncovered head. This produces a soothing effect, which keeps the little one fast asleep until the mother returns.

Should we find any of our neighbors' babies laying in the front yard, under the hydrants, after this issue of the Bistoury, we will be sorry we gave this information.

LIFE OF HORACE GREELEY.—The announcement that the writing of the life of Mr. Greeley is seriously contemplated—yea, that it has absolutely been consummated, ought to startle that gentleman and throw the public into a fever of expectation. We have been favored, by the author, with an opportunity for examining the manuscript of the work, and can best apprise our readers of the character of it, by quoting its title page, which runs as follows: "Life of Greeley—the Horse! Written through the Influence and Intrigue of Spiritual Mediums. Containing a Series of Wonderful Facts and Startling Revelations. Darwinian Theories Verified. The Veil Lifted and Slightly Tattered. Organization of The Democratic Party, or the Fall of Man.—Retrogration and Extinction of The Negro Race. Making the Only and Complete Life ever written of The Great Philosopher! Multum in Parvo, Six Volumes in One, by Prof. J. B. Rosewood." There, if any one can gaze upon such a title page without being seized with an irresistible desire to become acquainted with the mysteries of the book, they must be possessed of more self-control and complacency than is awarded to most human beings.

The work is to be profusely illustrated, is written in a Mark-Twain-sort-of-style, and is of course a broad burlesque upon Horace Greeley and the age. The author is a new candidate for literary honors, a physician, and resides in the neighboring village of Watkins.

NEW JERSEY STATE LUNATIC ASYLUM.—We have received a communication from Jos. S. COOK, M. D., censuring the managers of this institution, and complaining that he was wrongly and maliciously imprisoned, and badly treated for a period of one hundred and two days, by Dr. Buttolph, the Superintendent of the concern. We know nothing of the merits of the affair, and having heard only one side of the case, will make no further comments.

---

AN EGOTISTICAL ASS.—This unrare species of the animal kingdom, has a representative in a little town in Ohio. He is a contributor to, and writes nonsense for, the Philadelphia *Medical & Surgical Reporter*. He became seriously offended at our mailing clerk recently, and wrote a saucy, ill-natured, badly spelled and ungrammatical letter to the editor of this journal. We intended publishing his effusion in order that he might see how his articles appear before they are "fixed up" by the editor, and made readable. But upon inquiry, we learn that he is so universally regarded as an egotistical ass, at home, that no proof upon this point is needed. His signature reads: T. CURTIS SMITH, M. D., *Middleport, O.* Try again, Curtis.

---

NEW MEANS OF BURIAL.—An ingenious and thoughtful Frenchman, who has witnessed the unnecessary expense attending a fashionable funeral, has invented a process by which dead bodies can be liquified and transformed into a syrup, without color or smell. According to his calculation, a moderate sized man could be contained in a gallon jar. Now isn't that a splendid idea? What an opening for the exercise of filial piety, eh? When one's father dies, convert him into syrup, bottle him, and stand him on a shelf on the what-not in the parlor, labeled, "essence of father." Then, the third and fourth generations could add to the collection the essence of grandfather, great-grandfather, etc. Widowers could keep wife number one bottled for the inspection of wife number two, and it tickles one to think with what comical emotions wife number three would gaze upon the syrup of both her predecessors. By all means, let us have the French burial system; it is a cheap and decidedly unique process, and at once verifies the scripture, which we have somewhere read: "The dead have ye always with you." (Our friend H., who is better posted in biblical phrases than ourself, says that the quotation above made is not correct, in the word *dead*,—says it should be "poor." We can't spoil the item, however, to make such a trivial correction; if it isn't "dead," it *ought* to be—that's all).

---

INDIANAPOLIS.—We had occasion recently, to visit this beautiful and rapidly growing western city. Through the kindness of DAVID MCKERNAN and R. M. COFFIN, Esqs., we were shown the institutions of the city. Indianapolis is noted for its handsome public buildings—the Blind, Deaf and Dumb, Insane, and Inebriate Asylums; has also many elegant and costly mansions and beautiful business blocks, but the worst State House, poorest hotels, and the filthiest streets, we have ever seen. If the inhabitants in the vicinity of the Bates House alley, and the corner of Odd Fellows' Hall, escape cholera or dysentery this summer, it will not be because these localities have not invited such diseases by reason of the nastiness contained in their streets and gutters.

The city is supplied with water from an immense well, twenty feet in diameter and sixty feet deep, the propelling power being the "Holly Water Works" machinery. This is the most perfect system of water works yet devised, doing away entirely with fire engines on all streets where the water mains are laid, and supply the citizens with an abundance of clear, pure water. The cost of the works for a city of 200,000 inhabitants is less than one hundred thousand dollars, nearly as much as has been expended in this city for fire engines, and water works, deficient in supply, and affording water unfit, and absolutely unhealthy, for culinary purposes.

Indianapolis has two public parks affording its citizens with pure air, cool water and abundant shade. The grass is kept closely shorn, and the children are allowed to romp and play upon it, to their great delight and healthfulness. But neither of these grounds can compare in beauty and grandeur, with our own lovely Eldridge Park. Indeed, we doubt whether there exists a pleasure ground anywhere in the country, aside from Central or Fairmount Parks, that can compare with the elegant one furnished us through the munificence of our large-hearted fellow citizen, Dr. ELDRIDGE.

Somehow, we have got Indianapolis and Elmira so mixed up in this article, that it is difficult to tell which we purposed "noticing," so we will separate them by simply remarking that Indianapolis is a very beautiful city—with very many bad smells, but, that Elmira is the loveliest, and most cleanly little city to live in, that we have ever yet visited.

---

OURSELF.—Our readers are witnesses to the fact that we have never written ought of ourself or of the institution which we are conducting, or have made any reference to the same in the Bistoury. We hope therefore they will pardon us for publishing the article below, assuring them that we shall not occupy space for such a purpose again.

We are so nicely fitted up in our new establishment, and enjoy our new home so thoroughly, that we thought perhaps our friends would be glad to learn what sort of a place THE BISTOURY hails from. Mr. TABER, of the *Daily Advertiser*, having recently reconnoitered the place, and having been *shocked* at the display of certain electrical instruments, took occasion to revenge himself upon us in the following rather lengthy "local," which is sufficiently descriptive of our place:

THE ELMIRA EYE AND EAR INSTITUTE.—We made a visit to this establishment on Saturday, on a tour of inspection. We saw many strange and curious things. We were not aware that it was possible for the human frame to get so badly out of repair until we had made an inventory of the appliances necessary for correcting all sorts of deformities.

The doctor occupies eight handsomely furnished apartments in which he conducts his business. At the head of the stairs we were shown into the Ladies' and Gentlemen's waiting rooms, two spacious parlors on either side of the hall. Here, the patients who attend upon the morning and evening clinics, await their turn until summoned to the operating room by the ringing of a bell. At the time of our visit, all the patients were receiving treatment for diseases of the eye. We noticed that every patient was known by a number, which corresponded to a cup and brush on a large cabinet, kept for the purpose; from this he received the application to the eye and then departed, at the ringing of the bell, to make room for others. In this manner from forty to fifty patients, afflicted with eye difficulties are treated daily. Later in the day a surgical clinic is held, at which operations are performed for the cure of blindness, cross eye, crooked limbs, and for the removal of tumors, &c. The operating room is a wonderful place, fitted up with all manner of contrivances for the relief of injuries, and the cure of deformities and disease. The large instrument case occupying one end of the room is filled with beautiful surgical instruments of the most perfect workmanship—instruments so ingenious in construction and design that one is almost persuaded to submit to a surgical operation for the pleasure (?) of seeing the brilliant little things do their work.

Operating chairs, comfortable in upholstery and curious of machinery and shape, are also to be seen in this room, inviting the sufferer to an easy position under the surgeon's knife. Fracture beds that can be converted into chairs or traveling carriages, braces for curved spines, shoes for club foot, contrivances for contracted limbs, in short, all sorts of appliances for the cure of those suffering from accidents or deformity.

We were also shown the new process for removing highly vascular tumors, by means of the *galvano cautery.* A plantinum wire is placed about the tumor to be removed, fastened to an instrument called an *ecraseur*, and then connected by means of wires to an immense battery; the wire surrounding the tumor is brought to an incandescent heat, when the loop is gradually shortened, until the tumor is burned and lifted from its resting place. We saw a young man at the institute who had three tumors removed from his neck in this manner, and who declares the operation to be almost painless.

The examination room is perfectly dark, and fitted up with an *opthalmoscope*, by means of which the interior of the eye is illuminated and examined, enabling the observer to detect any disease of the humors of the eye, or of its retina or nerve. Also the *laryngoscope* for examining the throat, speaking apparatus, &c. But what astonished us most, was an electrical instrument for illuminating the interior of the abdomen, rendering the interior of the body so transparent as to enable the physician to detect the presence of abdominal tumors, enlargement of the liver, etc. Indeed, it is marvelous to see the ingenious contrivances with which the intelligent surgeon is equipped now-a-days, for discovering diseases of the human body. Dr. T. S. UP DE GRAFF has thousands of dollars invested in these curious and interesting devices, and it is a rare treat for any person who is fortunate enough to be allowed to examine them.

We spent a short time in the doctor's private study, looking over his handsome library and beautiful pictures. This is the cosiest room in the establishment, elegantly furnished, and open only to the doctor and his most intimate friends.

The labratory is a neat little room arranged and furnished purposely for compounding and dispensing medicines for the patients of the Institute, and is under the care of a competent pharmacist.

*The Bistoury*, the neat little magazine of which the doctor is editor, and which has secured so wide spread a circulation, also has a room for its accommodation, in which it is mailed and other business of its publication attended to. A neat little printing press is kept in this room, and is busy printing envelopes, blanks, notices of subscription due, &c., for the use of the magazine,

DR. UP DE GRAFF has practiced surgery in our city for the past ten years, and is perhaps as widely known as any surgeon in the State. Patients are found in his Institution from all parts of the United States, which is sufficient evidence of his popularity and success.

